# RURAL OLDER ADULTS’ VIEWS OF DEPRESCRIBING AND NONPHARMACOLOGICAL METHODS IN PAIN TREATMENT

**DOI:** 10.1093/geroni/igad104.2006

**Published:** 2023-12-21

**Authors:** Hyunjin Noh, Haelim Jeong, Denise Kan, Lewis Lee

**Affiliations:** University of Alabama, Tuscaloosa, Alabama, United States; The University of Alabama, Tuscaloosa, Alabama, United States; University of Alabama, Tuscaloosa, Alabama, United States; School of Social Work, The University of Alabama, Tuscalossa, Alabama, United States

## Abstract

Proper pain management is essential to quality of life. Due to the risks of harmful drug interactions, there has been an increasing effort to reduce medication that may be harmful or ineffective (i.e., deprescribing) and to promote non-medication based (i.e., non-pharmacological) strategies in pain management. The purpose of this qualitative study is to understand the views of deprescribing and non-pharmacological methods in pain management among community-dwelling older adults with multiple chronic health conditions. Eligibility criteria included: 65+, Alabama resident living outside of nursing homes, cognitively intact, have two or more chronic health conditions and chronic pain, and take medications for their health conditions including pain medications. Participants were recruited through the Area Agency on Aging across the state of Alabama as well as other community venues serving older adults. Individual, open-ended interviews were conducted by phone to explore their concerns about medications, their views of deprescribing and using non-pharmacological pain management as well as their needs in doing so. Thematic analysis of the interview data revealed various barriers and needs: concerns about various side effects while also worrying about missing out benefits from the medications if reduced or stopped, uncertainty or skepticism toward non-pharmacological pain treatment, lacking financial and logistical access to non-pharmacological options, and lack of knowledge about available non-pharmacological options and their benefits. These findings have implications for future education and advocacy efforts to promote older adults’ knowledge and self-efficacy so that they can consider deprescribing and non-pharmacological methods in managing their pain.

